# Lodestar DX, an evaluation: loop-mediated isothermal amplification (LAMP) for the diagnosis of urinary tract infection in symptomatic adult females

**DOI:** 10.1093/jacamr/dlae148

**Published:** 2024-09-17

**Authors:** Joanna Diggle, Ayako Van Der Goes Van Naters, Mohammed Ali Roula, Shanine Mitchell, Dewi Whiffen, Jeroen Nieuwland, Emma Hayhurst

**Affiliations:** Public Health Wales Microbiology, University Hospital of Wales, Heath Park, Cardiff CF14 4XW, Wales; University of South Wales, Faculty of Computing, Engineering and Science, Pontypridd CF37 1DL, Wales; Llusern Scientific Limited, Cardiff Edge Business Park, Longwood Drive, Cardiff CF14 7YU, Wales; Llusern Scientific Limited, Cardiff Edge Business Park, Longwood Drive, Cardiff CF14 7YU, Wales; University of South Wales, Faculty of Computing, Engineering and Science, Pontypridd CF37 1DL, Wales; Llusern Scientific Limited, Cardiff Edge Business Park, Longwood Drive, Cardiff CF14 7YU, Wales; Public Health Wales Microbiology, University Hospital of Wales, Heath Park, Cardiff CF14 4XW, Wales; Llusern Scientific Limited, Cardiff Edge Business Park, Longwood Drive, Cardiff CF14 7YU, Wales; University of South Wales, Faculty of Computing, Engineering and Science, Pontypridd CF37 1DL, Wales; Llusern Scientific Limited, Cardiff Edge Business Park, Longwood Drive, Cardiff CF14 7YU, Wales; Llusern Scientific Limited, Cardiff Edge Business Park, Longwood Drive, Cardiff CF14 7YU, Wales

## Abstract

**Background:**

Over 404.6 million people are affected worldwide each year by urinary tract infections (UTIs), with ∼237 000 associated deaths globally in 2019. Much more common in women than men, acute UTI occurs in up to 50% of the female population. Despite this, there is a lack of good diagnostic tools for use at the point-of-care, and over- and under-diagnosis are common, leading to long-term complications and patient suffering, and driving the spread of antimicrobial resistance through insufficient appropriate antibiotic stewardship.

**Objectives:**

To evaluate the performance of a novel point-of-care testing platform, Lodestar DX, in comparison with standard laboratory processing of urine specimens.

**Methods:**

A total of 199 fresh urine samples from symptomatic adult females suspected of having an acute UTI were tested using Lodestar DX and the results compared with standard laboratory methods performed at a local microbiology laboratory.

**Results:**

Using standard laboratory methods, 129/199 samples produced a result and could be compared. Overall sensitivity and specificity of Lodestar DX were 88.1% (95% CI: 77.8%–94.7%) and 83.9% (95% CI: 72.3%–92.0%), respectively (*n* = 129), with a positive predictive value of 85.5% (95% CI: 76.9%–91.3%), a negative predictive value of 86.7% (95% CI: 77.1%–92.6%) and an overall accuracy of 86.1% (95% CI: 78.9%–91.5%).

**Conclusions:**

The results show good correlation between Lodestar DX results and those of the standard laboratory method for this patient group. However, the platform would benefit from further testing to establish its true point-of-care compatibility and a direct comparison between this and other testing methods, such as urine dipstick testing.

## Introduction

More than 404.6 million people are affected worldwide each year by urinary tract infections (UTIs), with an estimated 237 000 associated deaths globally in 2019.^1^ As a result, UTIs place a significant burden on the NHS, being the second most common healthcare-associated infection in the UK after respiratory infections,^2^ and the leading cause of *Escherichia coli* bloodstream infections.^3^ UTIs are also responsible for approximately one-fifth of antibiotic prescriptions in the UK,^4^ making UTIs an important focus for improving patient outcomes and antibiotic stewardship. However, UTI remains a difficult infection to diagnose accurately, with many primary care settings adopting different strategies and decision making processes.^3^

A UTI is defined as an infection that invades any part of the urinary system, from urethra to kidneys, and is usually caused by bacteria from the gastrointestinal tract. If left untreated or misdiagnosed, UTIs have the potential to cause life-threatening illness such as sepsis.^5,6^

These infections are much more common in women than men, with acute UTI occurring in up to 50% of the female population.^7^ It is estimated^7^ that one-third of women will have experienced cystitis (acute UTI) at least once by the age of 24 and that approximately 30% of women will have a recurrent UTI within 3–4 months.^8^ Recurrent UTI can occur for decades in some women, and this condition has a considerable negative effect on women’s health and wellbeing, affecting the ability to sleep and exercise as well as the impact on sexual intimacy and mental health.^9^

The predominant cause of these UTIs is *E. coli*, a bacterium that accounts for approximately 80% of infections,^10^ followed by *Klebsiella pneumoniae*, *Enterococcus faecium* and other organisms. Many of these species are commonly associated with nosocomial infections and antibiotic resistance, and can be found on the WHO’s recent list of bacterial priority pathogens.^11^

### UTI diagnosis

For women under 65 years of age presenting with suspected UTI, the main diagnostic methods are empirical, dipstick and laboratory culture. Exact diagnosis varies between regions but in England, the number of key diagnostic symptoms present (dysuria, nocturia, cloudiness)^12^ is important to determine next steps. If two or three key symptoms are present, a dipstick is not needed and the clinician is advised to either watch and wait with a backup antibiotic, or to prescribe. If one key symptom is present, or no key symptoms but other urinary symptoms (such as urgency, frequency, haematuria or suprapubic tenderness), a urine dipstick test is performed and the results used to determine treatment. Dipsticks are designed to detect varying levels of proteins, blood, glucose, ketones, bilirubin and urobilinogen, and leucocyte esterase and nitrite.^13^ The result of the dipstick test is then used to determine next steps and treatment options. In certain circumstances, including atypical symptoms^14^ or the presence of one symptom and a mixed dipstick result,^12^ urine culture is recommended to confirm diagnosis.

However, the current guidelines may be inadequate, with one study finding that one in five women (20.4%) with two or more key symptoms do not have significant microbial growth when their urine is cultured, and 77% of those with no key symptoms do have significant growth.^15^ There is therefore significant scope to enhance current UTI diagnostics, improving both antibiotic stewardship and patient outcomes.

### Loop-mediated isothermal amplification

Developed in 2000 by Notomi *et al.*,^16^ loop-mediated isothermal amplification (LAMP) is a single-tube molecular technique for the amplification of nucleic acids under isothermal conditions (usually 60°C–65°C). This is a rapid diagnostic technique that has displayed high sensitivity and specificity from the outset and has already been applied in a number of fields such as medicine, agriculture and food industries. The process is simple, specific, fast, low-cost and highly compatible with point-of-care (POC) analysis, with potential to massively improve upon current POC diagnostic techniques.^17^

The Lodestar DX diagnostic platform, developed by Llusern Scientific Limited, is a device that runs and interprets the results of LAMP assays, giving users an indication of the presence or absence of specific pathogens. The assay evaluated in the current study is the Llusern Scientific LSL-HUTI UTI diagnostic test panel for six common uropathogens. The test takes 35 min, requires 10 µL of urine sample and requires no sample extraction prior to testing. Individual results are displayed on the Lodestar DX unit for each of the target pathogens on the panel, with a green light indicating a negative result and a red light indicating a positive result. According to local pathogen prevalence data gathered from Cardiff, UK in 2022, the LSL-HUTI panel should be able to detect approximately 87% of all UTI positives identified by culture method.^18^

### Objectives

- To conduct a clinical performance evaluation of the Llusern Scientific Lodestar DX diagnostic platform for the diagnosis of UTI in symptomatic adult females, suspected of suffering from an acute UTI.
- To quantify the sensitivity and specificity of the LSL-HUTI panel for UTI diagnosis both overall and for each specific target in the panel.

## Materials and methods

### Single assay optimization

Each individual assay was verified using between 45 and 180 urine samples (total = 511) to ensure calibration with relevant microbiological thresholds. Results from this work informed final sample preparation methodology for the full clinical performance evaluation.

### Analytical sensitivity

Prior to carrying out the clinical performance evaluation, the analytical sensitivity of each individual pathogen assay was determined. Serial dilutions of known copy numbers of target DNA were added to each assay in triplicate. Results were recorded and the lowest copy number of target DNA that produced a positive result in all three triplicate tests was used to calculate the limit of detection (LoD) of the assay. The copy number per reaction of the LoD was used to calculate the likely clinical threshold by converting to the corresponding cfu per mL.

### Clinical performance evaluation overview

Evaluation of the full UTI test panel took place independently of Llusern Scientific, with tests being carried out and results matched by Public Health Wales (PHW) to reduce bias, before being anonymized and shared with Llusern Scientific.

**Figure 1.**
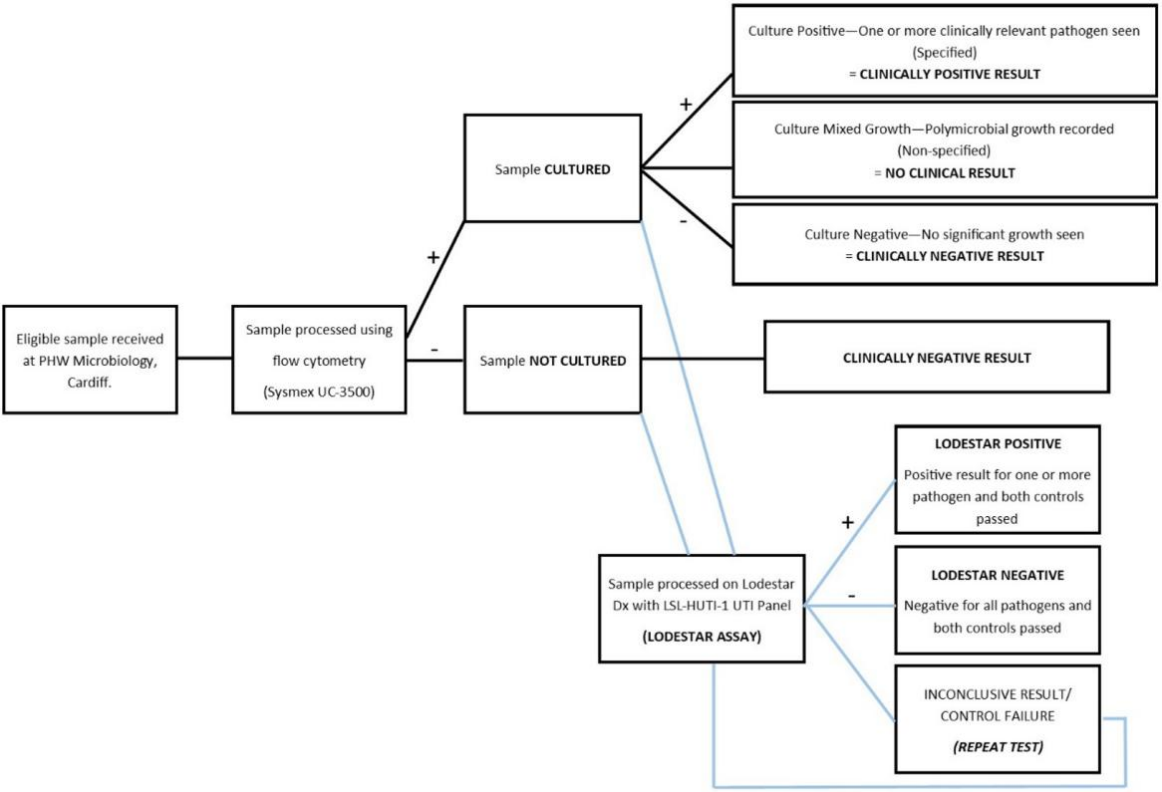
Clinical performance evaluation process overview.

Using the Llusern Scientific LSL-HUTI panel and the Lodestar DX diagnostic platform, a total of 199 fresh urine samples were tested and the results compared with those of the mainstream flow cytometry and culture method performed at PHW Microbiology, Cardiff. Target sample size was calculated, estimating a 50% prevalence and a target sensitivity and specificity of greater than 70%, as 200.^19^ Samples were processed and results recorded using the method displayed in Figure 1; all were tested within 4 h of receipt at the laboratory, prior to the availability of any culture result and regardless of whether or not the sample returned a positive or negative result by initial flow cytometry (Sysmex UF-5000) screening. These results were then further categorized as described in Table 1, to facilitate the generation of sensitivity and specificity percentages.

**Table 1.** Protocol for categorization of results

Result	Criteria
True positive	Positive for one or more pathogens by both Lodestar DX and culture
True negative	Lodestar DX negative and culture negative
False positive	Lodestar DX positive but culture negative
False negative	Lodestar DX negative but culture positive
Inconclusive	Control failure/LAMP inconclusive result/no clinical result available for culture (mixed growth sample)
Sensitivity %	True positives/(true positives + false negatives) × 100
Specificity %	True negatives/(true negatives + false positives) × 100

The LSL-HUTI panel consists of a strip of 8 × 0.2 mL PCR reaction tubes each containing a LAMP assay specific to each target on the panel (*E. coli*, *Enterococcus* sp./*Staphylococcus aureus* (combined) *Staphylococcus saprophyticus*, *Proteus mirabilis*, *Pseudomonas aeruginosa*, *Klebsiella pneumoniae*), a positive and negative control. A 10 µL sample of urine is added to a diluent tube, mixed, and then 5 µL is added to each pathogen assay and the positive control. Users receive a result indicating the presence or absence of each pathogen, allowing the potential causative agent of each UTI to be identified.

### Patient group

All 199 samples were selected from those sent from any care setting for routine urine culture to the PHW Microbiology laboratory at the University Hospital of Wales, Cardiff, and tested within 4 h of arrival at the laboratory. Ninety-six were from GP surgeries, 75 from outpatient clinics and 28 from hospitalized patients. The inclusion criterion was any urine sample from a symptomatic adult female suspected of having an acute UTI, without any of the exclusion criteria. Exclusion criteria were: any urine sample from an adult male; child (under 18); catheterized individuals; severely immunocompromised individuals; individuals suspected of having a yeast infection; and individuals suspected of having a chronic UTI. The decision on inclusion/exclusion of samples was made by J.D. based upon the clinical information provided on the test request (e.g. frequency, dysuria, abdominal pain). If no clinical information was given, a sample was not included.

### Reference method

The UTI diagnostic method employed by PHW involved initial processing of urine samples with a Sysmex UF-5000 flow cytometry urine chemistry analyser, followed by culture and EUCAST disc diffusion method for antibiotic sensitivity testing, for all samples designated with a positive Sysmex UF-5000 result. Local criteria for determining a positive Sysmex UF-5000 result at PHW Cardiff were as follows: >40 WBC or >250 bacteria.

Culture was performed using the ProLab PreLUD semi-automated culture platform by dispensing and spreading 10 µL of urine sample onto chromogenic UTI agar, by the calibrated loop method. One sample was allocated per agar plate; the plate was then incubated at 35°C–37°C for 16–24 h prior to being read and the results recorded. For this patient group, culture results are considered clinically significant if the growth observed is >10^6^ cfu/L (10^3^ cfu/mL). (Although PHW reports results in cfu/L, here they are reported in their equivalent cfu/mL, for consistency.) Organisms that required further identification after isolation with chromogenic media were subcultured onto Columbia blood agar and identified by Bruker MALDI-TOF.

This PHW Microbiology laboratory is UKAS accredited under ISO 15189, and all samples are processed in accordance with UK Standards for Microbiology Investigations (UK-SMI) 41i8.7.^20^ The standard results used for comparison in this evaluation were those from the routine NHS reports generated by this method.

## Results

### Single assay optimization

Results for each single assay optimization are shown in Table S1 (available as Supplementary data at *JAC-AMR* Online).

### Analytical sensitivity

The analytical LoD was determined for each individual assay and results are shown in Table 2.

**Table 2.** Analytical sensitivity of each individual assay, in copy numbers of target DNA per reaction, and the equivalent cfu/mL, based on existing methodology

Pathogen	Analytical sensitivity (number of copies of target DNA per reaction)^a^										LoD^b^	Equivalent cfu/mL
	500	250	125	83	63	50	25	12	8	6		
*E. coli*	+++	+++	+++	+++	+++	− − −	nt	nt	nt	nt	**63**	Between 10^4^ and 10^5^
*Enterococcus/S. aureus*	+++	nt	nt	nt	nt	+++	+++	−++	− − +	−++	**25**	Between 10^4^ and 10^5^
*S. saprophyticus*	+++	+++	+++	+++	+++	− − +	nt	nt	nt	nt	**63**	Between 10^4^ and 10^5^
*P. mirabilis*	+++	nt	nt	nt	nt	+++	−++	− − −	− − −	− − −	**50**	Between 10^4^ and 10^5^
*P. aeruginosa*	+++	nt	nt	nt	nt	+++	+++	−++	−++	− − −	**25**	Between 10^4^ and 10^5^
*K. pneumoniae*	+++	nt	nt	nt	nt	+++	+++	−++	−++	− − −	**25**	Between 10^4^ and 10^5^

nt, not tested.

^a^Analytical sensitivity is shown as positive (+) and negative (−) reactions at each dilution of target DNA, in triplicate.

^b^Limit of detection (LoD) (in bold) was determined as the lowest dilution for which all three triplicate samples showed a positive result and is shown in copy numbers of target DNA per reaction.

### Clinical performance evaluation

From the 199 samples tested, 59 true positive results were produced, along with 52 true negatives, 10 false positives and 8 false negatives (Table 3), some of which can be explained. The remaining 70 samples were designated inconclusive and were not included for evaluation, as described in Table 1. A full breakdown of these results can be seen in Table 4.

**Table 3.** Clinical performance evaluation results for Lodestar DX and LSL-HUTI test panel compared with laboratory methods (129 matched samples)

	Lodestar DX diagnosis of UTI		
Standard laboratory diagnosis of UTI	Positive	Negative	Total
Positive	59	8	67
Negative	10	52	62
Total	69	60	129

**Table 4.** Clinical performance evaluation results for Lodestar DX and LSL-HUTI test panel compared with laboratory methods (199 samples)

Full UTI panel results		
Result	Total (in bold)	
True positive		**59**
High concentration (>10^5^ cfu/mL)	29	
Medium concentration (10^4^–10^5^ cfu/mL)	29	
Low concentration (10^3^–10^4^ cfu/mL)	1	
True negative		**52**
False positive		**10**
False negative		**8**
Low concentration (10^3^–10^4^ cfu/mL)	1	
Uncommon pathogen	4	
Unknown cause	3	
Inconclusive		**70**
Mixed growth—Lodestar positive	19	
Mixed growth—Lodestar negative	50	
Inconclusive—result of unknown clinical significance	1	
Total		**1**

The 129 samples with evaluable results were used to calculate an overall sensitivity and specificity of 88.1% (95% CI: 77.8%–94.7%) and 83.9% (95% CI: 72.3%–92.0%), respectively. This gave a positive predictive value (PPV) of 85.5% (95% CI: 76.9%–91.3%), a negative predictive value (NPV) of 86.7% (95% CI: 77.1%–92.6%)^19^ and an overall accuracy of 86.1% (95% CI: 78.9%–91.5%).

Of the eight false negative results, one was from an *E. coli* sample with a recorded cfu/mL of <10^4^, which may be below the threshold for detection on Lodestar using current methodology. Three samples were *Enterococcus* sp. positive by culture and were not detected by Lodestar. All three had a recorded cfu/mL of between 10^4^ and 10^5^ and were reported as clinically significant by the laboratory. The other four were positive for pathogens that are not targeted by the LSL-HUTI panel, specifically, *Serratia marcescens*, *Citrobacter koseri*, *Streptococcus agalactiae* (Group B) and *Enterobacter hormaechei*.

Of the 59 true positive results, 29 were from samples with a recorded cfu/mL of >10^5^, 29 were from samples with a recorded cfu/mL of >10^4^–10^5^, and 1 was from a sample with a recorded cfu/mL of >10^3^–10^4^.

A total of 70 samples returned an inconclusive result due to the prevalence of non-specified polymicrobial/mixed growth samples by culture, which could not be interpreted given there was no clinical result available for comparison. Of those 70 samples, 51 also returned a negative result on the Lodestar, but the remaining 19 samples produced a positive result for one or more clinically relevant pathogens.

Eight samples returned a Sysmex UF-5000 negative sample but were positive in Lodestar. All of these were cultured but only one grew a significant amount of bacteria (*E. coli*; >10^3^ cfu/mL). The clinical significance of this result was unclear, so the sample was removed from analysis. The remaining samples that were Sysmex UF-5000 negative but Lodestar positive were recorded as false positives.

Of the 59 true positives detected, 45 were *E. coli*, which accounted for 76% of all true positives observed in the evaluation. Nine were *K. pneumoniae*, accounting for 15%, four were *S. saprophyticus* and one was *P. mirabilis*. No *Enterococcus* sp., *S. aureus* or *P. aeruginosa* true positives were detected in this dataset.

When considering the performance of the *E. coli* assay, the sensitivity and specificity were 97.8% (95% CI: 88.5%–99.9%) and 90.4% (95% CI: 81.9%–95.8%), respectively. Full details can be seen in Tables 5 and 6. This gave a PPV of 84.9% (95% CI: 74.4%–91.6%), an NPV of 98.7% (95% CI: 91.5%–99.8%) and an accuracy of 93.0% (95% CI: 87.1%–96.8%).^21^

**Table 5.** Clinical performance evaluation results for Lodestar DX and the *E. coli* assay compared with laboratory methods (129 matched samples)

Standard laboratory diagnosis of *E. coli*	Lodestar DX diagnosis of *E. coli*		Total
	Positive	Negative	
Positive	45	1	46
Negative	8	75	83
Total	53	76	129

**Table 6.** Clinical performance evaluation results for Lodestar DX with the individual *E .coli* assay, compared with laboratory methods (199 samples)

Full UTI panel results (*E. coli* ONLY)		
Result	Total (in bold)	
True positive		**45**
True negative		**75**
False positive		**8**
False negative		
Low concentration (10^3^–10^4^ cfu/mL)		**1**
Inconclusive		**70**
Mixed growth—LAMP positive	8	
Mixed growth—LAMP negative	62	
Total		**199**

## Discussion

### Performance of the Lodestar DX testing platform for the diagnosis of UTIs

The analytical LoD for all assays in the UTI test panel was between 10^4^ and 10^5^ cfu/mL, which corresponds to many laboratories’ growth threshold to indicate UTI in the UK.^12^ However, in some patient populations (strongly symptomatic women, men^12^) and in other geographical locations,^14^ the UTI threshold is lower. The sensitivity of Lodestar’s UTI test panel can be increased significantly by making small changes to the sample preparation methodology, but whether all assays in the panel could reliably detect samples with 10^2^–10^3^ cfu/mL requires further investigation. In this sample set, only 2 out of 67 (3%) culture-positive samples had a cfu lower than 10^3^/mL. The vast majority (97%) had bacterial concentrations above Lodestar’s analytical LoD.

The overall sensitivity and specificity of the LSL-HUTI panel were 88.1% (95% CI: 77.8%–94.7%) and 83.9% (95% CI: 72.3%–92.0%), respectively. This gave a PPV of 85.5% (95% CI: 76.9%–91.3%) and an NPV of 86.7% (95% CI: 77.1%–92.6%). However, the PPV and NPV of this study may not be truly reflective of the clinical situation at POC because of two factors: (i) the inclusion of mixed growth samples as either ‘positive’ or ‘negative’ would affect disease prevalence, and (ii) there were likely to be more patients with UTI symptoms who did not have their urines sent for culture, and who would therefore not be included in this study. If they were included, disease prevalence would be less, altering PPV and NPV values.

When considering the performance of the *E. coli* assay, the sensitivity and specificity were 97.8% (95% CI: 88.5%–99.9%) and 90.4% (95% CI: 81.9%–95.8%), respectively, with a PPV of 84.9% (95% CI: 74.4%–91.6%), an NPV of 98.7% (95% CI: 91.5%–99.8%) and an accuracy of 93% (95% CI: 87.1%–96.8%). Individual results for the other assays in the panel could not be reported from this evaluation due to the low prevalence or absence of these pathogens within the sample set. Single assay optimization work showed good performance of all assays (Table S1) but, as sample preparation methodology has been altered since this work, further study to evaluate the performance of these assays is necessary before conclusions can be drawn.

A recent systematic review of the clinical effectiveness of UTI POC tests identified four rapid (<40 min) tests either on or close to market in the UK, one of which was Lodestar DX.^22^ Only two had any published sensitivity or specificity values—Uriscreen’s sensitivity ranged from 61% to 89% across four studies, and UTRiPLEX’s only study concluded a sensitivity of 21% and a specificity of 94%. However, these were evaluated with differing patient groups and therefore are not directly comparable to this particular evaluation and chosen patient group.

There were three false negatives for *Enterococcus* in the evaluation. Two of those had only *Enterococcus* on the culture plate, the third was mixed growth with *Enterococcus*. All had a cfu/mL of between 10^4^ and 10^5^, which may have been below the limit of detection for this assay. Alternatively, these may have been from a non-target species as they were not identified to species level by the laboratory. Further analysis of this assay is needed to determine its performance.

There were 10 false positives in total. These may have been samples where bacterial DNA was present but no viable bacteria. For example, this could occur if the patient was already taking antibiotics.

### Mixed growth samples

Although urine culture is the globally accepted laboratory standard, UTI diagnosis remains clinically challenging. Single bacterial growths with significant colony counts are considered diagnostic, but polymicrobial growth is usually considered contamination, even though 30%–39% of UTIs are true mixed infections.^23^ They are usually reported as more than three colony types with a specified predominant type, and a request can be made for a repeat sample with proper collection process.^23^ However, in roughly one-third of all the samples reported in this dataset, no predominant organisms were identified, and no clinical result was obtained, meaning a significant proportion of samples could not be compared and no clinical result was achieved.

It is likely that contamination was affecting some of these samples, but considering that all of the samples tested were from symptomatic patients, at least some were likely to be true infections. In these cases, especially where clinical information also suggests a true infection, it is important to correctly identify the pathogens in order to streamline therapy^23^ and prevent delayed treatment and additional patient suffering.

Of the 70 samples that returned a mixed growth culture result, a positive result was returned on 19 samples using Lodestar DX. However, the clinical significance of these results is unclear. Clearer interpretation of polymicrobial growth results is hypothesized to impact clinician’s treatment decisions.^24^ To understand this and the effectiveness of both Lodestar and culture in polymicrobial UTI diagnosis is an important future aim.

### Inhibition and troubleshooting

Throughout the evaluation, only eight (4%) tests failed and needed to be repeated due to unexplained negative control failures, with the vast majority passing first time and returning an acceptable result.

A further seven (3.5%) of the sample’s positive control failed resulting in the test having to be repeated. This was due to a known issue recognized through prior testing, of inhibition caused by the presence of either extremely high RBCs or high WBCs in the sample. This inhibition is detected by adding sample to the positive control. If the positive control fails it is assumed the test is being inhibited by something in the sample and therefore must be repeated.

This inhibition occurred in one sample that had extremely high RBCs of over 40 000 ×10^6^/L in the Sysmex UF-5000 result. This was overcome by diluting the sample 1:2 prior to retesting. The other six failed tests were presumed to be caused by high WBCs in the sample (>2000 ×10^6^/L), which again were either overcome by diluting at a ratio of 1:2 or by simply allowing the urine sample to settle to allow the formation of sediment to occur prior to repeating the test from the clearer fluid at the top of the tube.

### Potential impact

The results in this study indicate that Lodestar DX has a high NPV, particularly for *E. coli.* This may improve antibiotic stewardship by giving prescribers the information they need to make evidence-based treatment decisions. Some primary care practitioners have suggested that an accurate POC test to aid in the diagnosis of a UTI would be very useful in order to reassure the patient, and to reliably determine the need for antibiotics.^3^ This is likely to result in the reduction of antibiotics prescribed for UTIs and provide a cost saving for healthcare providers. Prescribing antibiotics unnecessarily not only increases the risk of resistance, it can also increase an individual’s risk of developing recurrent UTI.^14^

### Conclusions

The need for a rapid POC test for the diagnosis of UTIs has been widely recognised.^22,25^ The results in this study show good correlation between Lodestar DX and those of the standard laboratory method for acute UTI in adult females, suggesting that Lodestar DX may have potential as a diagnostic tool to provide clinicians with further evidence to make their treatment decisions.

Limitations of the study include the lack of patient information written on the clinical report for some samples and the problem of comparing Lodestar results with those of standard laboratory methods—different laboratories use different standard methods for UTI diagnosis, so the results in this study may not be generalizable. Sample size was also smaller than expected due to the large number of excluded mixed growth samples, giving wider 95% CIs.

Going forward, the test would benefit from a larger scale evaluation at near patient settings to give a more accurate indication of its performance in real-world settings. The evaluation could also be extended to other patient populations including males, children, and complicated and recurrent UTI, using appropriate thresholds of infection. More investigation is also needed into the clinical significance of mixed growth samples and the diagnostic capabilities of Lodestar for these. Comparison with other available POC UTI diagnostic tools, including dipstick and symptoms, and the relative impact on antibiotic stewardship and patient outcomes would help determine Lodestar’s potential to improve diagnosis of this important infection.

## Supplementary Material

dlae148_Supplementary_Data

## Supplementary data

Table S1 is available as Supplementary data at *JAC-AMR* Online.
