# Supplementary material for: Lodestar DX, an evaluation: loop-mediated isothermal amplification (LAMP) for the diagnosis of urinary tract infection in symptomatic adult females
Source: JAC Antimicrob Resist. 2024 Sep 17;6(5):dlae148. doi: 10.1093/jacamr/dlae148 (PMC11406545; doi:10.1093/jacamr/dlae148)
Supplement: dlae148_Supplementary_Data [file dlae148_supplementary_data.docx]

**Supplementary Data**

| **Target Pathogen** | **Sample size** | **TP** | **TN** | **FP** | **FN** | **Initial sensitivity (%)** | **Initial specificity (%)** | **No. of FNs inhibited by high WBCs** | **Potential improved sensitivity (%)** |
| --- | --- | --- | --- | --- | --- | --- | --- | --- | --- |
| *E.coli* | 180 | 33 | 62 | 3 | 15 | 68.8 | 95.4 | 11 of 15 | 91. |
| *K.pneumoniae* | 103 | 56 | 29 | 3 | 15 | 78.9 | 90.6 | 13 of 15 | 97.2 |
| *Enterococcus/*  *S.aureus* | 73 | 33 | 19 | 3 | 9 | 78.6 | 86.4 | 1 of 9 | 81.0 |
| *P.mirabilis* | 55 | 15 | 19 | 0 | 4 | 79.0 | 100.0 | 3 of 4 | 94.7 |
| *P.aeruginosa* | 45 | 19 | 15 | 1 | 3 | 86.4 | 93.8 | 1 of 3 | 90.9 |
| *S.saprophyticus* | 55 | 10 | 27 | 0 | 1 | 90.9 | 100.0 | 0 of 1 | n/a |

**Supplementary Table One**. Single-assay optimisation and verification. Each individual pathogen assay was tested using a combination of fresh and stored urines. The number of FN samples potentially inhibited by high WBC count was recorded, and once WBC inhibition issues were solved by altering the sample preparation methodology, the potential improved sensitivity (assuming the new methodology would mean FN samples with high WBC would no longer be inhibited and would become TPs) was calculated.
